# Optogenetic Control of Transcription in Zebrafish

**DOI:** 10.1371/journal.pone.0050738

**Published:** 2012-11-30

**Authors:** Hongtao Liu, Gustavo Gomez, Sophia Lin, Shuo Lin, Chentao Lin

**Affiliations:** 1 National Key Laboratory of Plant Molecular Genetics and National Center for Plant Gene Research (Shanghai), Institute of Plant Physiology and Ecology, Shanghai Institutes for Biological Sciences, Chinese Academy of Sciences, Shanghai, China; 2 Department of Molecular, Cell and Developmental Biology, University of California Los Angeles, Los Angeles, California, United States of America; Indian Institute of Science, India

## Abstract

Light inducible protein-protein interactions are powerful tools to manipulate biological processes. Genetically encoded light-gated proteins for controlling precise cellular behavior are a new and promising technology, called optogenetics. Here we exploited the blue light-induced transcription system in yeast and zebrafish, based on the blue light dependent interaction between two plant proteins, blue light photoreceptor Cryptochrome 2 (CRY2) and the bHLH transcription factor CIB1 (CRY-interacting bHLH 1). We demonstrate the utility of this system by inducing rapid transcription suppression and activation in zebrafish.

## Introduction

Using the combination of genetic and optical methods to control specific events in targeted cells or organisms has allowed recent development of optogenetics technology [1]. In comparison to other methods used to manipulate cellular functions and processes, optogenetics methods offer certain advantages, such as rapid delivery, lack of toxicity, and reversibility.

Light-dependent protein-protein interaction is a convenient approach in optogenetic control of cellular functions. For example, a phytochrome-dependent transcription regulatory system has been reported for the light control of gene expression in yeast. Phytochromes are plant red/far-red light photoreceptors that undergo red light-dependent physical interaction with the basic helix-loop-helix proteins PIFs (phytochrome interacting factors). Phytochromes and PIFs have been used as the dimerizer pair to make the red light controlled system, such as the light switchable transcription system in yeast [2]. In addition, other approaches, such as the light regulated protein translocation system have also been widely used [3]. Although the interaction between Phytochromes and PIF offers rapid stimulation and reversibility, the interaction requires a bilin cofactor which could not be found in lots of organisms, such as animals, including zebrafish. This deters the use of this system in other organisms that do not synthesize the bilin cofactor.

Cryptochromes (CRY) are photolyase-like photoreceptors that regulate growth and development in plants and the circadian clock in plants and animals. Plant cryptochromes are best studied in the reference plant Arabidopsis. Arabidopsis CRY1 and CRY2 mediate primarily blue light regulation of de-etiolation and photoperiodic control of flowering, respectively. The cryptochrome protein contains two domains, the N-terminal PHR (Photolyase-Homologous Region) domain of about 500 residues, and the C-terminal extension CCE (Cryptochrome C-terminal Extension, also called CCT) of various lengths. PHR is the chromophore-binding domain of cryptochromes that binds non-covalently to the chromophore flavin adenine dinucleotide (FAD) and possibly a second chromophore, 5,10-methenyltetrahydrofolate (MTHF) [4], [5], [6]. The CCE domain of Arabidopsis CRY1 and CRY2 which functions as an effector domain is approximately 180 and 110 residues in length, respectively. Arabidopsis CRY2 undergoes blue light-specific interaction with the bHLH protein CIB1 (CRY-interacting bHLH 1), which was isolated in a blue light-differentiated yeast-two-hybrid screen [7]. CIB1 is the first protein that interacts with CRY2 in a blue light specific manner in plant, and is a transcription factor whose activity is also blue light and CRY2 dependent.

The chromophore of cryptochromes, FAD, is synthesized in all organisms. The CRY2-CIB1 interaction can be triggered at a subsecond time scale, and is reversible within minutes [8], making it an attractive optogenetics system. A blue light triggered protein translocation system and a DNA recombination system in living cells were made based on the blue light triggered interaction of CIB1 and CRY2 [8]. Very recently, optogenetic control of phosphoinositide metabolism was also developed based on the CIB1 and CRY2 pair [9]. There are blue light inducible transcription systems in yeast and plants [7], [8], but so far no artificial light inducible transcription system has been reported in a vertebrate organism. Here we describe a blue light inducible transcription system in zebrafish.

**Figure 1:**
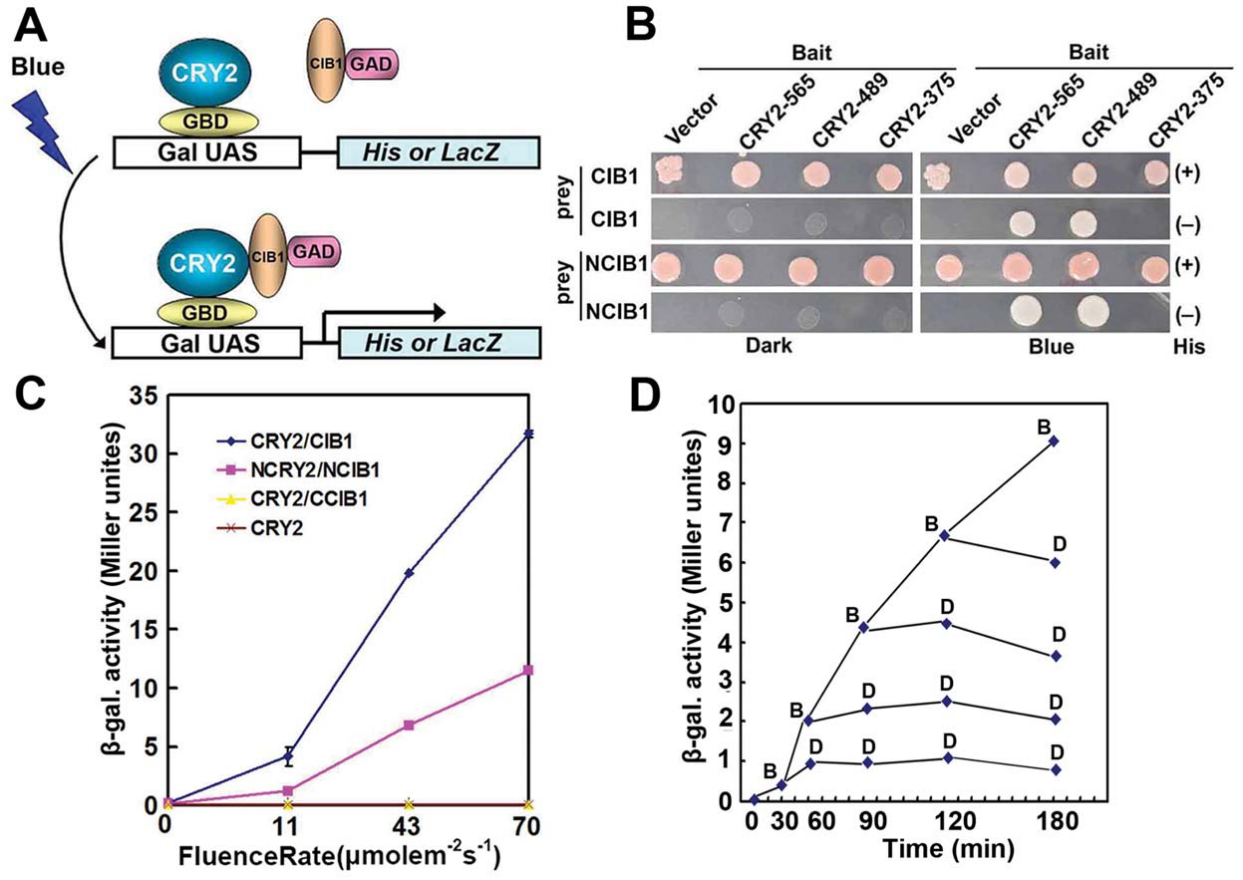
Blue light-dependent transcription regulation in yeast. A. Schematic of split Gal4 modules expressed in yeast cells containing His or LacZ reporter genes under control of a galactose-inducible promoter. In the dark the reporter gene is off. To induce expression of the reporter gene, cells are exposed to blue light which triggers the interaction of CRY2 and CIB1, so that the reporter gene gets activated. B. Histidine auxotrophy assays showing blue light-dependent interaction between CRY2N-489 or 565 and CIB1 or CIB1 N171, and the lack of interaction between CRY2N-375 and CIB1 or CIB1 N171. Yeast cells containing plasmids encoding the indicated proteins were grown on medium in the presence (+) or absence (−) of histidine, under blue light (Blue, 30 µmolm^−2^ s^−1^) or in the dark (Dark) for 3 days. C. β-Gal assays of yeast cells expressing indicated proteins irradiated with different fluence rate of blue light (0 to 70 mmol m^−2^ s^−1^) for 60 minutes. D. Effect of dark treatment in reversing induction of gene expression by blue light treatment. Yeast cells co-transformed with BD-NCRY2 and AD-NCIB1 were grown in the dark first for 2 hour, then moved to blue light (20 mmol m^–2 ^s^–1^ ) for 30,60,120,180 min, at every time point, yeast were either incubated further in the blue light for the periods indicated (B) or moved back to dark for the periods indicated (D). For example, when the yeast were treated with blue light for 30 min, samples were taken for the β-galactosidase assay, then the yeast cells were split into two part, one was kept in the blue light condition, while the other one was put into dark condition, 30 min later, samples were take from both the blue light treated and the dark kept yeast cells for the β-galactosidase assay.

**Figure 2:**
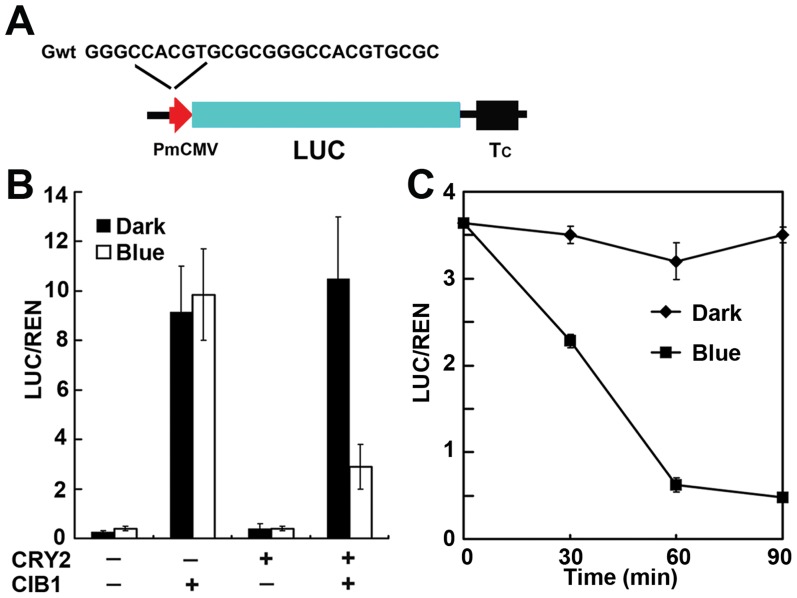
Blue light-suppressed transcription regulation in zebrafish. A. Structure of the G-box–driven LUC reporter gene and DNA sequences of the recombinant G-box (Gwt). CMV minimum promoter (red arrow head), firefly luciferase (LUC), are indicated. B. Relative reporter activity (LUC/REN) in zebrafish expressing indicated genes (CRY2 and CIB1). Zebrafish embryos were injected with the reporters (LUC and REN control) and the effectors (CRY2 and CIB1), kept in dark for 3 hours, then some were irradiated with blue light (20 mmol m^–2 ^s^–1^) for 2 hours (Blue) while some were kept in dark (Dark). The relative LUC activities normalized to the REN activity are shown (LUC/REN). The experiment was repeated 3 times with pools of 30 embryos per condition each time. C. Relative reporter activity (LUC/REN) in zebrafish expressing CRY2 and CIB1 in dark or blue light. Zebrafish embryos were injected with the reporters and the effectors (CRY2 and CIB1), kept in dark for 3 hours, then some were irradiated with blue light (20 mmol m^–2 ^s^–1^) for the periods indicated (Blue) while some were kept in the dark (Dark). The relative LUC activities normalized to the REN activity are shown (LUC/REN, n = 3).

## Materials and Methods

### Yeast Two Hybrid

Experiments using the yeast two-hybrid system are as described (7), and/or according to the manufacturer’s instructions (Matchmaker user’s manual, Clontech, California). The coding sequences of CRY2, CRY2N565 which contains the residues 1 to 565, CRY2N489 (residues 1 to 489) and CRY2N375 (residues 1 to 375) were fused in-frame with the GAL4 DNA binding domain (BD) of the bait vector pBridge (Clontech). The coding sequences of CIB1 and CIB1N171 which contains the residues 1 to 171 were fused in –frame with the GAL4 AD domain of the prey vector pGADT7 (Clontech). The bait plasmids and the prey plasmids were co-transformed into the yeast strain Y190. To analyze CRY2 and CIB1 interaction by the histidine auxotrophy assay, yeast colonies were patched in duplicate onto His−/+3AT and His+ plates. One duplicate was grown under blue light (30 µmolm−2s−1), at 28°C, for 2–3 days. The second duplicate was wrapped in aluminum foil to block the light, and grown at the same condition. For the β-galactosidase assay, yeast cells were grown overnight in SD medium, diluted 5 folds in YPD medium, grown in the dark for 1–2 hr, transferred to light of different wavelengths and fluence rates (or left in the dark as controls), and grown for up to 190 min, during which samples were taken for the β-galactosidase assay.

### Dual LUC Assay in Zebrafish Embryos

The reporter plasmids, pGAL4.20-G-LUC and pGAL4.20-BD-LUC encode the firefly luciferase controlled by the recombinant G-box promoter or the GAL4 promoter, and the *Renilla* (REN) luciferase controlled by the constitutive SV40 promoter was used as an internal control. The recombinant G-box promoter, which contains two copies of the wild-type G boxes (CACGTG) fused to the minimum SV40 promoter, was cloned into the XhoI/Hind? sites of the vector pGAL4.20, the GAL4 promoter was cloned into the KpnI/XhoI sites of pGAL4.20.

Reporter DNA was microinjected into zebra fish’s embryos with or without MycCIB1, VP16CIB1N-GAD mRNA or CRY2, CRY2N-GBD mRNA. The fish embryos were left under 30°C in the dark for 3 hours, one aliquot were treated with blue light (20 µmolm−2 s−1) while another aliquot was kept in dark for another 1 to 2 hour. The dual-luc assay using the commercial Dual-LUC reaction reagents was used to test the transcription activity of the reporter promoter according to the manufacturer’s instruction (Promega). Briefly, 10 to 20 embryos were freeze in liquid nitrogen, and homogenized in 40 µl of the Passive Lysis buffer (Promega). 8 µl of the crude extract was mixed with 40 µl of Luciferase Assay buffer (Promega), and the firefly luciferase activity (LUC) was measured, using a luminometer (tunor 20/20, Promega). After measurement of the firefly luciferase activity, 40 µl of the Stop and Glow buffer (Promega) was added to the reaction to quench the firefly luciferase and to initiate the REN luciferase (REN) reaction. Three biological repeats were measured for each sample.

**Figure 3:**
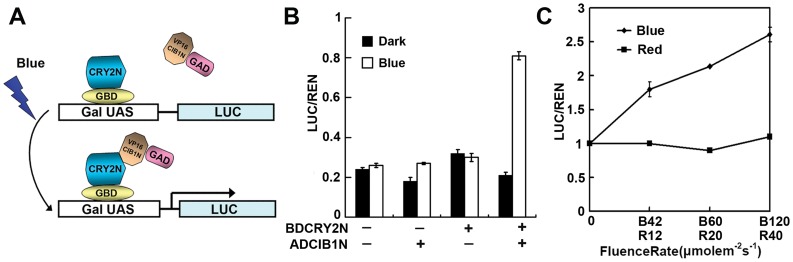
Blue light-activated transcription regulation in zebrafish. A. Schematic of split Gal4 modules expressed in zebrafish together with a LUC reporter gene under control of a galactose-inducible promoter. B. Relative reporter activity (LUC/REN) in zebrafish expressing indicated genes (BD-CRY2N and AD-CIB1N). Zebrafish embryos were injected with the reporters and the effectors (BD-CRY2N and AD-CIB1N), kept in dark for 3 hours, then some irradiated with blue light (20 mmol m^–2^ s^–1^) for 2 hours (Blue) while some were kept in the dark (Dark). The relative LUC activities normalized to the REN activity are shown (LUC/REN). The experiment was repeated 3 times with pools of 30 embryos per condition each time. C. Relative reporter activity (LUC/REN) in zebrafish embryos expressing BD-CRY2N and AD-CIB1N in red or blue light. Embryos were injected with the reporters and the effectors (BD-CRY2N and AD-CIB1N), kept in dark for 3 hours, then some were irradiated with different fluence rate of blue light (42 to 120 20 mmol m^–2 ^s^–1^) for 2 hours (Blue) while others were irradiated with different fluence rates of red light (12 to 40 20 mmol m^–2 ^s^–1^) for 2 hours (Red). The relative LUC activities normalized to the REN activity are shown (LUC/REN, n = 3).

## Results

### Blue Light-dependent Transcription Regulation in Yeast

We developed a blue light inducible transcription system based on the yeast two hybrid concept in yeast (Fig. 1A). We first mapped the interaction domains for the blue light dependent CRY2-CIB1 interaction using the yeast two hybrid assay. The N-terminal photolyase homology region (PHR) of CRY2 is sufficient to mediate blue light dependent interaction with CIB1. Amino acid 375–489 of CRY2 contains the CIB1 interaction domain, while the 171 N-terminal amino acids of CIB1 mediates the interaction with CRY2 as shown by two different reporter assays, the histidine auxotrophy (Fig1 B), and the β-galactosidase (β-gal) activity (Fig. 1C). In yeast cells, both full length CIB1 and the N terminal of CIB1 interact with NCRY2-565 (CRY2 N terminal 565 amino acids), NCRY2-489 (CRY2 N terminal 489 amino acids) only in blue light but not in the dark, as demonstrated by the blue light-dependent rescue of *His3* transcription and histidine auxotrophy (Figure 1B). In contrast, yeast cells expressing CIB1 or NCIB1 with NCRY2-375 (CRY2 N terminal 375 amino acids) failed to rescue the histidine auxotrophy either in blue light or dark (Figure 1B), indicating that NCRY2-375 can not interact with CIB1. As shown in Figure 1C, no β-gal activity was detected in cells expressing only CRY2 or CRY2 and C terminal 164 amino acid of CIB1 (CCIB1), even under the highest fluence rate. In contrast, cells expressing both CRY2 and CIB1 or NCRY2 (CRY2 N terminal 489 amino acid) and NCIB1 (CIB1 N terminal 171 amino acid) showed a fluence rate-dependent increase of β-gal activity after normalization by cell number. The interaction of NCRY2 and NCIB1 is not only fluence rate-dependent but also duration-dependent, as cells irradiated with blue light of the same fluence rate but for a longer duration also exhibited higher β-gal activity (FigS1). The blue-light dependent interaction of CRY2 and CIB1 is rapidly reversed by dark treatment [8]. To determine whether dark could be used to reverse the induction of β-galactosidase activity in yeast, yeast cells were first grown in the dark, exposed to blue light, then the blue light induced cells were moved back to dark at either 30, 60, 90, 120, or 180 minutes. The results indicate that the dark treatment blocked any further increase in β-galactosidase activity beyond that reached at the time of the dark transition (Fig. 1D).

### Blue Light-suppressed Transcription Regulation in Zebrafish

We used a dual luciferase reporter system [7] under the control of the G-box (CACGTG)-containing promoter to test whether CRY2 can mediate light regulation of CIB1 activity in zebrafish. The reporter constructs, Gbox-LUC, which encodes the firefly luciferase controlled by the recombinant G-box promoter (it contains two copies of the wild-type G boxes fused to the minimal CMV promoter), and internal REN luciferase control, were microinjected into zebrafish embryos with or without MycCIB1 mRNA or CRY2 mRNA. Embryos were incubated at 30°C in the dark for 3 hours, one sample (about 30 embryos) was illuminated with blue light (20 µmol m^−2^ s^−1^) while the other was kept in the dark. Our result shows that CIB1 can act as a transcription activator of the recombinant G-Box promoter in this assay system. Importantly, the results shown in Fig. 2B demonstrate that CRY2 mediates blue-light dependent suppression of the CIB1 activity (Fig. 2B, compare Dark to Blue in the presence of both CRY2 and CIB1). We further tested the CRY2-mediated blue-light suppression of the CIB1 activity. Embryos expressing both CRY2 and CIB1 were incubated under blue light (20 µmol m^−2^ s^−1^) or in the dark for different time periods. As shown in Figure 2C, embryos that remained in the dark showed no change in expression even for the longest duration (90 min). In contrast, embryos exposed to blue light showed time-dependent suppression of LUC expression. Embryos exposed to blue light for the longer duration of irradiation exhibited higher suppression, suggesting a more robust interaction of CRY2 and CIB1 under longer duration of irradiation. We conclude that Arabidopsis CIB1 can act as a transcriptional regulator in zebrafish and cryptochrome can mediate blue light suppression of the CIB1 activity. Therefore, the CRY2-CIB1 pair can act as a blue light-dependent transcriptional regulator in a vertebrate organism.

### Blue Light-activated Transcription Regulation in Zebrafish

We then tested the CRY2-mediated blue light regulation of the activity of a different promoter, GAL4, using smaller fragments of CRY2 and CIB1, CRY2N489 and CIB1N171 (Fig. 3A). We prepared two constructs, CRY2N-GBD, which encodes the fusion protein of CRY2N489 fused to the GAL4 DNA-binding domain, and VP16CIB1N-GAD, which encodes the fusion protein of VP16 (a very strong transcription activation motif) fused to CIB1N171 first then fused to the GAL4 activation domain. We expressed CRY2N-GBD and VP16CIB1N-GAD in zebrafish embryos together with a reporter (LUC) under control of the GAL4 promoter, as well as the REN internal control. Embryos were incubated at 30°C in the dark for 3 hours, then illuminated with blue light (20 µmol m^−2^ s^−1^) for 2 hour or kept in the dark for two more hours, or kept in dark, and harvested at 5 hours post injection. The expression of the blue light inducible system and the blue light treatment did not dramatically affect the embryos’ development (FigS2). Dual LUC assay revealed a strong LUC expression in response to blue light when CRY2N-GBD and VP16CIB1N-GAD were expressed together. No LUC changes were detected when only one of these two partners was expressed (Fig. 3B). To test the wavelength specificity of this transcription activation system, zebrafish embryos expressing CRY2N-GBD and VP16CIB1N-GAD were irradiated with blue light or red light, and the LUC activity of the samples were analyzed after 30 minute of irradiation (Figure 3C). The result of this experiment shows that transcription is activated only by blue light but not by red light. For example, embryos irradiated with blue light (42 µmol m^−2^ s^−1^) showed an appreciable increase of LUC activity (Figure 3C, B42), whereas no LUC activity change was detected in cells irradiated with red light (40 µmol m^−2^ s^−1^) (Figure 3C, R40). The blue light dependent transcription is also fluence rate dependent, since cells irradiated with higher fluence rate of blue light exhibited higher LUC activity (Figure 3C, compare B42 with B60 and B120).

## Discussion

The interaction of NCRY2 and NCIB1 is blue light dependent and it is fluence rate-dependent in yeast, the same as full length CRY2 and CIB1, indicating that NCRY2 and NCIB1 could be used as a blue light dependent interaction pair.

Our results also indicate that Arabidopsis CIB1 can act as a transcriptional regulator in zebrafish and cryptochrome 2 can mediate blue light suppression of the CIB1 activity. Therefore, the CRY2-CIB1 pair can act as a blue light-dependent transcriptional regulator in a vertebrate organism. Later we also made this blue light induced transcription system into a transcription activation system by genetic engineering both components of the dimer pair. To achieve this, the DNA binding domain and the transcription activation domain (bHLH domain) of CIB1 was deleted, only the CRY2 interacting domain-CIB1N171 was kept, and fused to the strong activation motif VP16 and the AD domain of GAL4. This VP16CIB1N-GAD can not work as a transcription factor by itself, since it only has the transcription activation domain. In like manner, the CRY2N terminal domain was fused to the BD domain of GAL4, converting it into a DNA binding protein. When the two fusion proteins were expressed together in zebrafish embryos, these two proteins can interact with each other in the blue light condition, and only when they interact with each other, they will work as a transcription factor, and the GAL4AD domain plus the VP16 transcription activation motif make the complex a transcription activator.

Taken together, our experiments demonstrate that the plant photoreceptor CRY2 can be used with its signaling partner CIB1 to generate a blue light-dependent transcription suppression system or a blue light-dependent transcription activation system in a vertebrate organism. We expect this plant-derived photoreceptor system may be a useful optogenetic tool for studies in animals.

## Supporting Information

Figure S1 **NCIB1 interact with NCRY2 in a fluence rate dependent manner.** β-Gal assays of yeast cells expressing CRY2N489-GBD and CIB1N171-GAD irradiated with different fluence rate of blue light (0 to 75 mmol m^−2^ s^−1^) for different duration (90 to 190 minute).
(TIF)

Figure S2 **Zebrafish embryos injected with the blue light inducible gene expression system.** Uninjected or embryos injected at the one cell stage with reporters only, VP16CIB1N-GAD (cib1) and reporters, CRY2N-GBD (cry2) and reporters, or VP16CIB1N-GAD plus CRY2N-GBD and reporters were imaged at time of harvest for dual luciferase assays.
(TIF)
