# Supplementary figures and images for: Optogenetic Control of Transcription in Zebrafish
Source: PLoS One. 2012 Nov 30;7(11):e50738. doi: 10.1371/journal.pone.0050738 (PMC3511356; doi:10.1371/journal.pone.0050738)

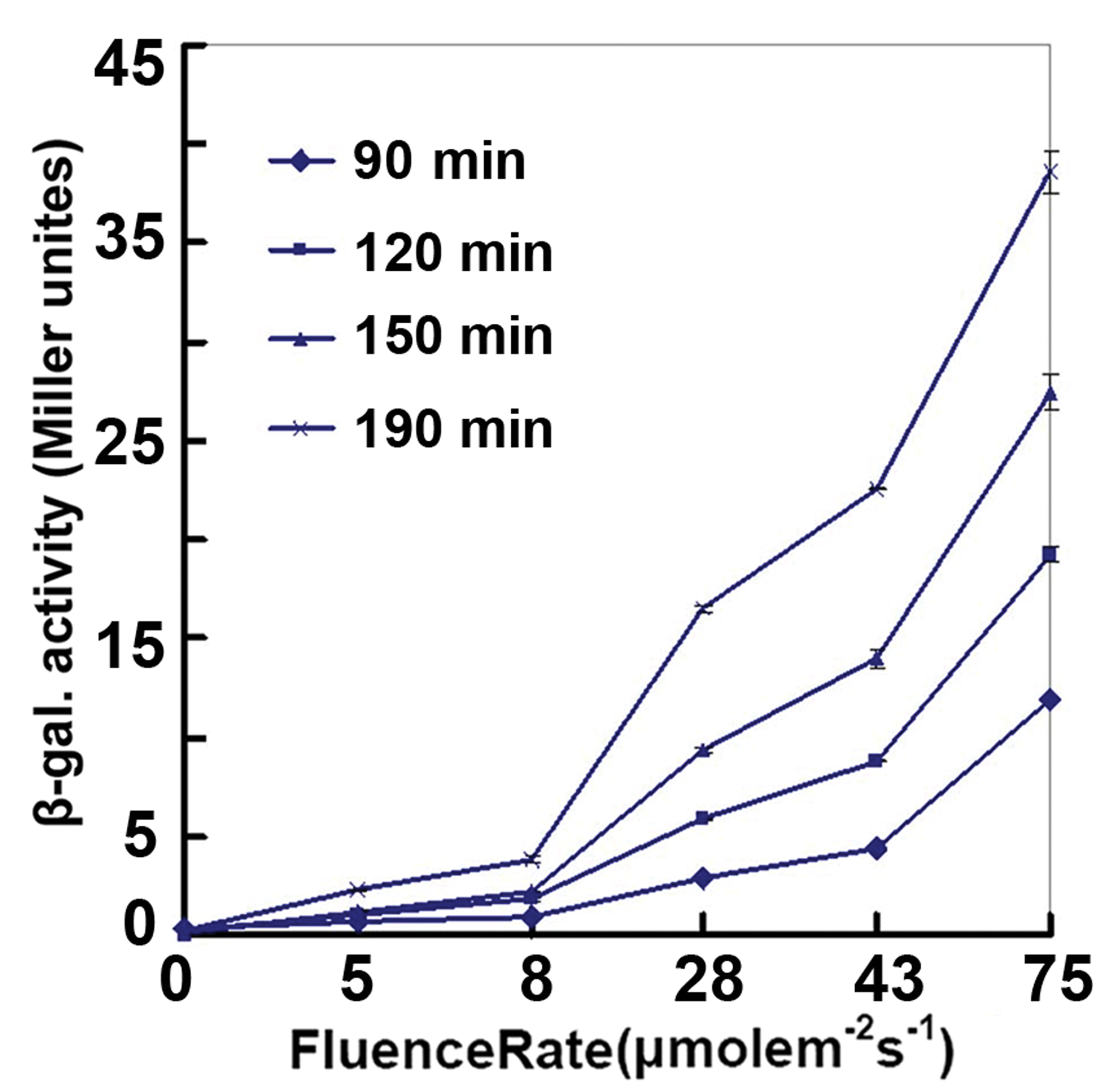

Supplement: Figure S1 — NCIB1 interact with NCRY2 in a fluence rate dependent manner. β-Gal assays of yeast cells expressing CRY2N489-GBD and CIB1N171-GAD irradiated with different fluence rate of blue light (0 to 75 mmol m−2 s−1) for different duration (90 to 190 minute). (TIF) [file pone.0050738.s001.tif]

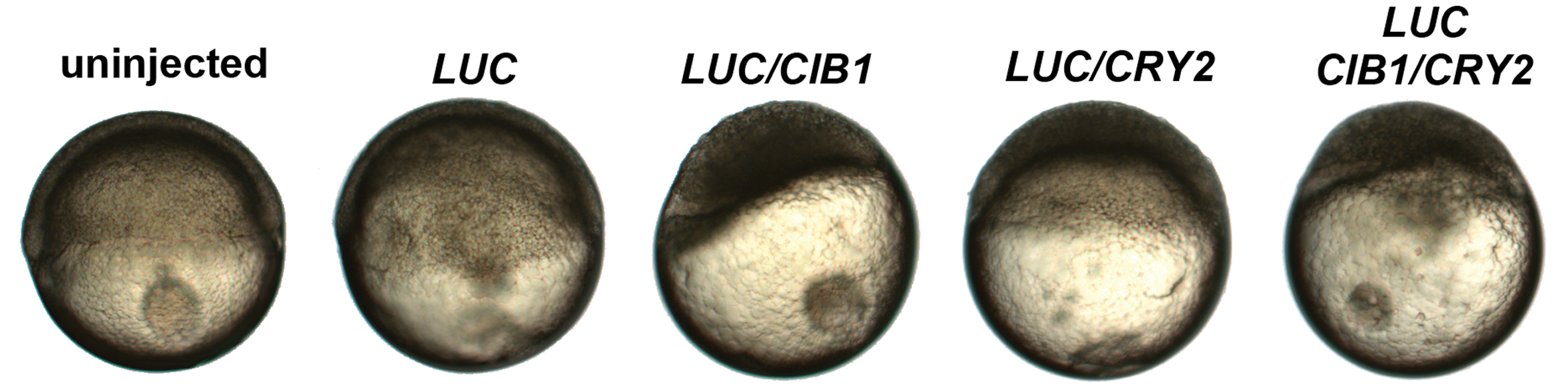

Supplement: Figure S2 — Zebrafish embryos injected with the blue light inducible gene expression system. Uninjected or embryos injected at the one cell stage with reporters only, VP16CIB1N-GAD (cib1) and reporters, CRY2N-GBD (cry2) and reporters, or VP16CIB1N-GAD plus CRY2N-GBD and reporters were imaged at time of harvest for dual luciferase assays. (TIF) [file pone.0050738.s002.tif]
